# Visual analysis of environmental research progress in Germany linking Development Anthropology: A sustainable approach based on the Web of Science

**DOI:** 10.3389/fpsyg.2022.1018183

**Published:** 2023-01-04

**Authors:** Wei Zong, Fei Wang, Bo Tu, Huazhong Tu, Hao Zhang, Chuke Wu, Muhammad Toseef

**Affiliations:** ^1^School of Ethnology and Sociology, Minzu University of China, Beijing, China; ^2^South Asian Studies Center, Institute of Area Studies, Honghe University, Mengzi, China; ^3^Institute of Area Studies, Honghe University, Mengzi, China; ^4^Business Administration, Silla University, Busan, South Korea; ^5^Baize Institute for Strategic Studies, Southwest University of Political Science & Law, Chongqing, China; ^6^School of History and Culture, Sichuan University, Chengdu, China; ^7^Faculty of Management & Economics, Kunming University of Science and Technology, Kunming, China; ^8^Department of Management Sciences, University College of Zhob (BUITEMS), Zhob, Balochistan, Pakistan

**Keywords:** economic growth, sustainable environmental, sustainable thoughts, Germany, Belt and Road, development anthropology

## Abstract

Environmental devaluation is a major concern for European countries as they seek to scrutinize strategies for development anthropology. Germany holds diversified ties with the socioeconomic and environmental development of the region. In accordance with global obligations, Germany's research on environmental issues, protection laws and actions, and universities and scientific research institutions in the field of environmental protection are progressing toward the development of a sustainable future securing the development anthropology. However, Germany's research on environmental issues is unclear to the rest of the world. Chinese scholars also lack effective countermeasures and suggestions for implementing environmental protection cooperation between China and Germany under the Belt and Road Initiative to draw a sustainable global drain. Understanding the current situation and frontier trend of environmental research in German academic circles is essential and irreplaceable for relying on research results data and quantitative analysis theory to carry out the research process. The methodology of this paper established a quantitative analysis based on “institutions,” “scholars,” “research objects,” and “frequency of keywords” among the research results on environmental issues in Germany. It constructs a digital portrait of the field of environmental research in Germany. Knowledge mapping is extensively used in this study as the primary research tool to show the development of environmental research in Germany. The standard deviation of social science research has roughly doubled in that time. CiteSpace, a visual tool for document statistical analysis, is used to analyze the research results on environmental protection published by German scholars from 2008 to 2018. The study results include Web of Science Network, and finally, a visual map is drawn. This study analyzes the status quo, research institutions, keywords, research hotspots, and research trends of international cooperation in environmental research in Germany. The findings are in supportive position of environment study that is the key to human existence and societal development. Leading to this Germans are in concern of human anthropology being reflected in scholarly published work. In response to practical challenges, “global warming” and “sustainable development” became the most frequently used keywords. It provides sustainable thoughts and countermeasures to strengthen Sino-German environmental protection exchange and cooperation further.

## Introduction

Germany is the most important European country and a sustainable engine for promoting economic growth. Germany has attached great importance to environmental protection the overall. Not only does the German government emphasize the significance of environmental preservation, but also all political parties, organizations, universities, scientific research institutions, and citizens place a high value on environmental protection as a national priority.

German mainstream media such as Süddeutsche Zeitung, Der Spiegel, Frankfurter Allgemeine Zeitung, “reported the connectivity of environment policies in promulgating developmental roots by the means of anthropology as a key to environment and societal norms” Rojas (2013). In various environmental protection demonstrations organized by the German Green Party in recent years, it was found that the German Green Party plays a particularly prominent role and often takes radical measures to appeal for environmental protection, which has caused widespread concern in Germany and abroad. The international community usually attaches great importance to environmental protection in Germany. According to Stone (2003) Germany's environmental protection policies provide a foundation to the anthropological field in designing societies. On 11 December 2019, the European Commission published the European Green Trade and its roadmap. Adjusting the European Union's climate change policy will help control climate risks and achieve green development in Europe and the rest of the world. China also attaches importance to green development. The concept of green sustainable development advocated by the Belt and Road Initiative is in line with the European Green New Deal. China hopes to promote sustainable economic development through the “Green” Belt and Road Initiative through green cooperation with Germany. Therefore, it is important to know more about Germany's research on environmental issues, be familiar with relevant environmental protection policies and measures, and know the focus of German universities and scientific research institutions on environmental protection and the progress made. There is great potential for Sino-German green cooperation in the future. Collaboration with a big country like Germany will also encourage China to actively achieve the goal of a carbon dioxide emission peak in 2030 and carbon neutrality in 2060 and contribute actively to building a community of shared future for mankind.

Most western countries were significantly impacted after the global financial crisis broke out in 2008. To avoid relying too much on the United States for their economic development, some European countries put their main efforts into the fields of new technology development, advanced equipment manufacturing, environmental protection, and so on, hoping to bring new growth points to their own economy. As the most important engine of economic growth in Europe, Germany has also been greatly affected since the global financial crisis broke out in 2008 (Rauh, 2010; Michael, 2021). The German government, universities, research institutes, and ordinary Germans have spared no effort in studying environmental problems. The vast investment in environmental research allows Germany great achievements in environmental research (Siebert, 2015). Lean and innovative approaches in SMEs in India are seen as responsive in terms of product. In fact, processes are inevitable in environmental performance (Shashi et al., 2019).

Environmental gains are the roots that vary in economic setup by means of CO_2_ emissions and the ultimate costs of technologies like blockchain (Centobelli et al., 2021; D'Acierno et al., 2022). As mentioned above, Germany not only promoted the economic growth of the European Union but also played a crucial role in promoting the development of global science and technology innovation (Thomas, 2017; Zhou, 2020). Compared with Germany, which attaches great importance to its own environmental problems at all levels of society, the outside world is not clear about international cooperation, cooperation between scientific research institutions, cooperation networks of researchers, cited networks, research hotspots, and research frontiers in German environmental research (Leach, 2013). It is also worth exploring how Chinese scholars can carry out environmental cooperation between China and Germany and take more effective countermeasures and suggestions. Leadership is pivotal for developing sustainable organizations to operate in a safe environment (Niemtu et al., 2022; Toseef et al., 2022). For future generations to benefit from current efforts, sustainable development is an instrumental tool for the current generation to make an optimistic use of resources (Barnes et al., 2013). Biodiversity and climate change pose a global risk to humanity and require serious action. Industries, universities, politics, economists, and leadership have the authority to redesign policies to remix economic, social, and environmental policies to redesign development into sustainable development. The study at hand took roots from the “Brundland model” proposed by the World Commission on Environment and Development (WCED) (1987). The model operationalized the term “sustainable development” and is commonly called “our common future.”

Furthermore, development is critical for all global partners to generate and consume resources without compromising the future generation. Scholars construct sustainability differently from an economic perspective to discover and consume the resources to attain development. The ecological model also requires the world to be materialistic. Environmental security through the means of biological diversity and ecological integration objectively replicates human, culture and economical anthropology (Wirzba, 2003; Agyeman, 2005; Norton, 2005; Jenkins, 2008; Leach, 2013; Zhou and Xue, 2016).

German academics and professionals working in universities and institutes have rectified the country's strategic policy on environmental issues through their research. Researchers in the country are also under pressure from the international community to develop innovative environmental solutions to slow down the effects of climate change. Policies are structural in shaping simple strategies along with the financial mechanism and target objectives. The territorial policies led to the developmental processes toward environmental sustainability (EC Quality of Public Administration, 2017; Medeiros et al., 2022). China is transforming up to the agenda 203 transformations of Chinese industrial development given by the United Nations for sustainable development, “transforming our world.” The Chinese government is promoting sustainable industrialization in connection with infrastructural development, technological and scientific innovation, energy conservation, and emission reduction (Guo and Hu, 2019; Zheng, 2020). Scholarly works in well-known journals is critical for developing globally accepted research work that promulgates the dominance of environmental studies in the ecological domain. In the environmental domain of Germany, the methodology of this study is based on knowledge mapping using CiteSpace software, connecting scientific literature to showcase trends and developmental issues being studied by scholars.

### Research question

Academicians and research institutes are Germany's leading forces in environmental protection research. This study's foundational questions are as follows: Did Germany synthesize a tree of international cooperation on environmental protection measures? Do universities and research institutions cultivate environmental research in Germany? Does China's Belt and Road project include European collaboration on environmental protection?

## Materials and methods

The qualitative and quantitative insights of the environmental research integrates previous studies under the literature-reviewed approach. Many existing studies offered a multidimensional view of the study topic, but limitations have been found in academic research. This led this study to bridge this gap with a literature review-based approach to analyze previously published work on environmental research in Germany. Bibliometric analysis emerged in the nineteenth century as an independent subject and evolved into a critical literature analysis tool-kit of the given field of study under quantitative methodology (Pritchard, 1969; De Bellis, 2009; Diem and Wolter, 2013; Mayr and Scharnhorst, 2015). Graphs, visuals, and network maps are the fruits of technology to complement existing literature. Previous studies stapled bibliometric analysis to visualize a vast field of research on brand personality (Llanos-Herrera and Merigo, 2018), E-health (Müller et al., 2018), social media (Li et al., 2017; Noor et al., 2020), computer-human behavior (Mou et al., 2019), blockchain wave and future of decentralized technologies (Centobelli et al., 2021), and other.

Many types of software exist in the application of bibliometric analysis. City Space is Java-based analytic software to produce tables and visual maps (Chen, 2004). In this study, a quantitative visualization technique is used by CiteSpace software to lay down network maps of countries, institutions, authors, and keywords related to environmental research using the 10-year (2008–2018) Web of Science database. The significance of bibliometric analysis using CiteSpace depicts visual clusters to draw multidimensional connectivity of studies on environmental research in Germany. In this study, the researchers used steps like material collection: bibliometric database selection; scope: preprocessing research tools and data cleaning; and result analysis: descriptive and visualization of environmental research trends in Germany. In English and journals, several research queries with relevant keywords are employed in the abstract, title, and author.

### Material collection

#### Bibliometric database selection

**Figure 1:**
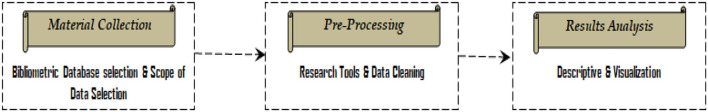
Time-shift diagram of high-frequency keywords and research frontiers of environmental problems in Germany.

At present, there are many databases in international academic circles, mainly including Scopus, Jstor, Taylor and Francis Online, and Web of Science. Among many databases, the Science Network contains the most data, covers the widest range, and has the most expansive influence. The latest published related research results reflect the international community's current research frontiers. Currently, some databases, such as Scopus, Jstor, or Taylor and Francis Online, are limited. There is either an interface problem with CiteSpace, the research tool chosen by this study, or the data volume is too small to meet the analysis goal of large samples. Thus, the author did not select Scopus, Jstor, or Taylor and Francis Online when deciding upon a database. Because most German scholars mainly use English for research in international academic exchanges and international cooperation and because the Web of Science covers the fields and topics that this research can cover, the author finally chose the Web of Science as the database source for data screening (see Figure 1).

#### Scope of data collection

After the global financial crisis broke out in 2008, the German government made a series of adjustments to its domestic and foreign policies, increased its investment in environmental research, and achieved good results in domestic environmental research. Based on the above analysis, the author took 2008 as the breakthrough point, selected the research results of German environmental problems from 2008 to 2018, and further analyzed the international cooperation, research institutions, keywords, research hotspots, and research trends of German environmental problems in the past 10 years to develop ideas for China's environmental protection and further strengthen the future exchanges and cooperation between China and Germany in environmental protection. Notably, although Germans are patriotic, they do not reject the English language in the process of international exchanges, cooperation, and scientific research. Most of their main achievements have been included in the Web of Science with the international association of German scholars in environmental research. Combined with the author's research theme and the characteristics of the Web of Science database, the author mainly included the research achievements of German scholars in English from the Web of Science, which reflected the research achievements of Germany. Therefore, in the data collection process, the author effectively reflects the basic situation and research frontier of German environmental research from the aspects of time choice and language choice, which can also answer the author's research questions. In the data collection process, he chose to set the scope of literature language retrieval to English, which does not affect the scientific and representative nature of this research.

### Preprocessing

#### Research tools

In recent years, with the continuous development of science and technology, especially the rapid development of computer science and technology, more and more scholars are using advanced research tools for quantitative research. With the continuous emergence of visual research tools, these scientific research tools are used to conduct quantitative research, which makes for the hot issues of important research frontiers and their international cooperation, research institutions, keywords, research hotspots, research trends, etc., which are concerning some countries at present and more vivid, making the research results more intuitive and academic, and more academic. Therefore, in this study, the author chose CiteSpace as the first tool for research and followed up with the visual analysis and operation of the research on the surrounding environment in Germany.

#### Data cleaning

After the author entered the page of the Web of Science TM core collection database, it mainly contains high-level research results, which can effectively reflect the latest research results of current international academic circles. First, the author limited the keyword to “environmental issues,” added the search criteria of “language,” set it to “English,” and then searched. The data screened out in the first round were 82,471. The disciplines cover many disciplines, such as environmental science, physics, ecology, economics, microbiology, computer science and artificial intelligence, public management, political science, history, with a solid interdisciplinary nature and wide coverage. The data covers periodical documents, conference papers, book reviews, data papers, secretary chapters, etc. Then, the author limited the time range to “2008–2018.” After the second round of cleaning up irrelevant data, the remaining valid data were 57,231, which covered the corresponding research results on environmental problems in various countries in the world in the last 10 years, which are included in the Web of Science TM core collection database. More than 100 other irrelevant countries included in the Web of Science TM core collection database must be excluded from the data to further show the relevance. By choosing “Germany” for further refinement, the author excluded other irrelevant countries such as the United States, Britain, and France; the remaining effective number is 2,714. The 2,714 valid data after the final data cleaning contained the research data on German environmental problems, which can truly and effectively meet the research goals set by the author.

## Results analysis

### Descriptive and visualization

CiteSpace is a Java-based research tool. Before the visualization operation, the authors needed to configure Java environment variables before starting the subsequent operation. After configuring the Java running environment, the author started the following related operations. It should be pointed out that CiteSpace was updated to its latest version. After downloading and installing CiteSpace, the author collected and cleaned data before starting subsequent operations. The author set the time to 2008–2018, and the time slicing was initially set to 1, which was later adjusted as needed in the subsequent analysis, which helped analyze the data more intuitively.

#### Analysis of scientific research cooperation

In the “Selection Criteria” functional area, the author set the TOP N to 50, thus selecting the top 50 nodes with the highest frequency in the time slice. After the aforementioned basic parameters were set, the subsequent operations were developed for different clustering nodes and thresholds according to different analysis requirements and then corresponding visual analyses according to the results. The cluster node was set to the country in the CiteSpace operation interface to analyze international cooperation in environmental research. The thresholds (c, cc, ccv) of the three time periods were assigned as (2, 2, 20), (4, 3, 20), and (4, 3, 20), respectively.

**Figure 2:**
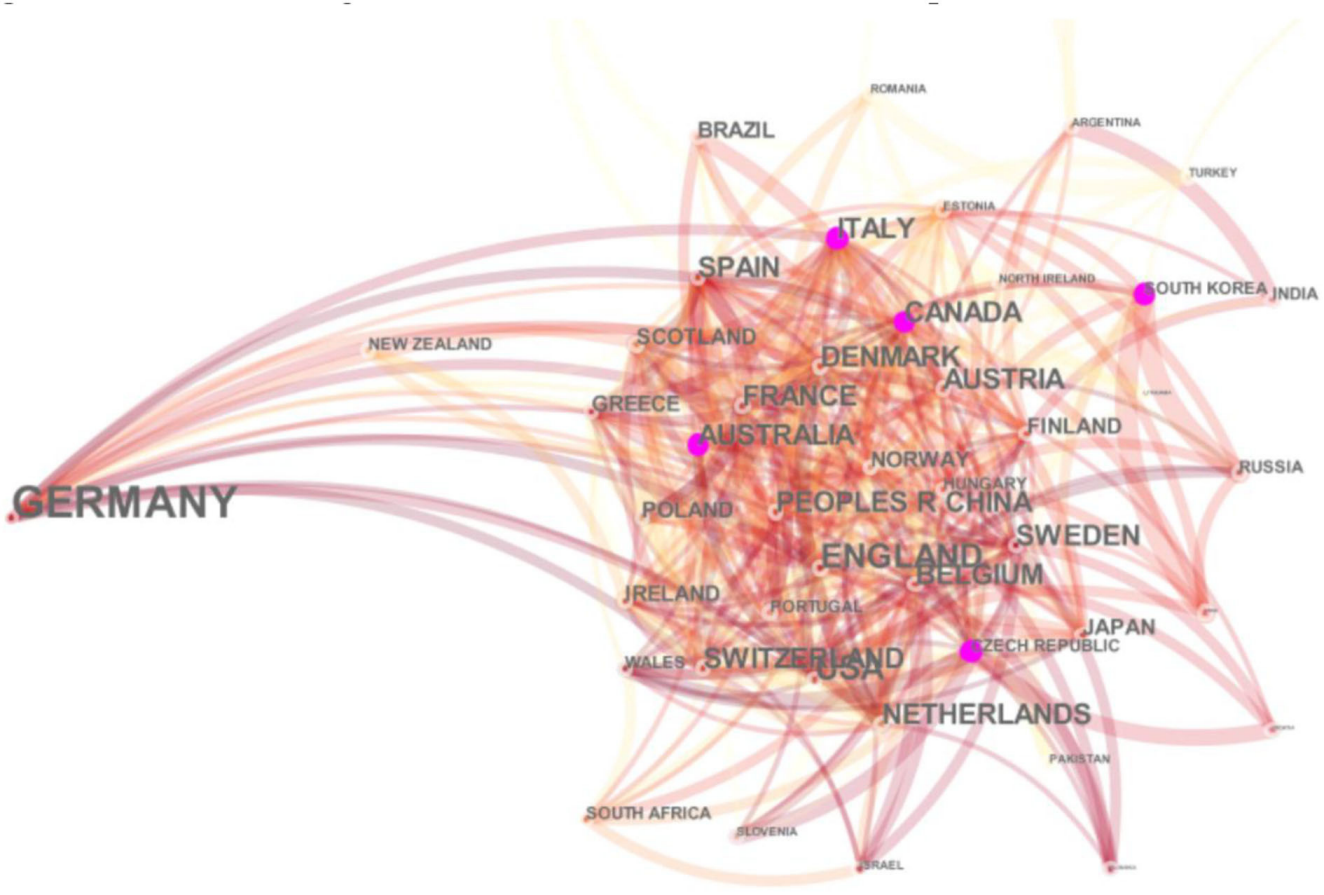
Schematic diagram of the German international cooperation network in environmental research. Source: This map was drawn by the author through CiteSpace.

We selected the “country” option in the CiteSpace interface, and the visualization results showed that there were 78 effective nodes and 727 links. From 2008 to 2018, Germany made 727 contacts with 78 countries worldwide (Figure 2).

**Figure 3:**
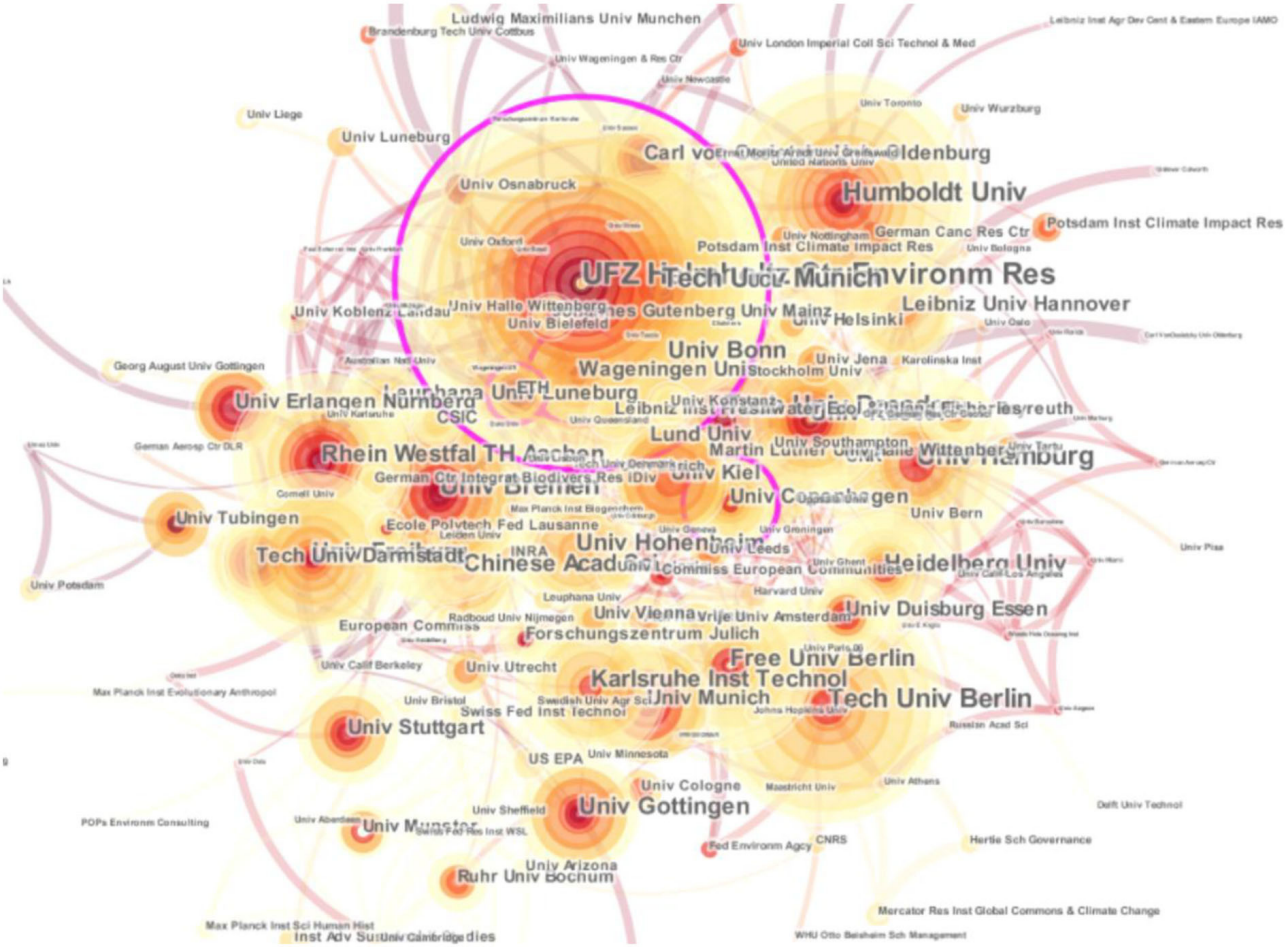
Environmental research cooperation network of German universities and research institutions. Source: This map was drawn by the author through CiteSpace.

Table 1 was found that Germany's international cooperation in environmental research was mainly led by German universities and think tanks, with a frequency of cooperation of 2,695 in German universities and research institutions, ranking first. German universities and research institutions cooperated extensively with research institutions in other countries and regions worldwide. These countries were the United States, Britain, Italy, France, the Netherlands, Switzerland, Spain, Sweden, Australia, Canada, China, Denmark, Belgium, Austria, Finland, and other countries and regions. Among them, Germany has the closest cooperation with American universities and think tanks, with 402 cooperations between the two countries, followed by the United Kingdom, with 321 cooperations between the two countries, focusing on China and Germany. The said results reflect the global collaboration of Germany through the contribution of universities, where academicians were selecting, investigating, and reporting environmental contributions across global partners. Secondly, the country's institutions also reported environmental facts to draw conclusive solutions and protective measures as responsible environmental stakeholders (see Figure 3).

**Table 1:** Fifteen countries, led by Germany, participated in international cooperation in environmental research.

**Serial number**	**Country**	**Frequency**
0	Germany	2,695
1	United States	402
2	United Kingdom	321
3	Italy	231
4	France	229
5	Netherlands	218
6	Switzerland	202
7	Spain	156
8	Sweden	152
9	Australia	130
10	Canada	129
11	China	126
12	Denmark	114
13	Belgium	110
14	Austria	106
15	Finland	82

Source: This form was drawn by the author through CiteSpace.

#### Analysis of cooperation among scientific research institutions

To further analyze the cooperation network between German universities and scientific research institutions, the author chose the cluster node “institution” in the CiteSpace operation interface and set the thresholds (C, cc, ccv) to (2, 2, 20), and (4, 3, 20), respectively. The Table 2 descriptive results highlight the depth and breadth of the research conducted on German universities and the significance of the resulting cooperation relations in environmental research. Germany stands at the top of the list with 2,695 participants in environmental research cooperation; the US, 402; UK, 321; Italy, 231; France, 229; Netherlands, 218; Switzerland, 202, and the rest of the remaining countries had < 200 participants, including China with 129 global environmental research projects.

**Table 2:** Top ten universities and research institutes engaged in environmental research in Germany with the highest frequency.

**Serial number**	**Organization**	**Frequency**
1	The Helmholtz-Centre for Environmental Research	99
2	Humboldt University	70
3	Technical University Dresden	69
4	Technical University Berlin	62
5	Technical University Munich	61
6	University Bremen	59
7	University Hamburg	55
8	University Bonn	50
9	RWTH Aachen University	49
10	University Freiburg	49

Source: This form was drawn by the author through CiteSpace.

Then, we selected the “institution” option, and the visualization results showed that there were 192 effective nodes and 594 links; that is, from 2008 to 2018, German universities and scientific research institutions, as well as German scientific research institutions and universities and scientific research institutions from other nations, collaborated on environmental research projects. The research status of German scientific research institutions more clearly and further highlights the research achievements of some German institutions. The author further screened out the data of German universities and scientific research institutions that participated in inter-agency cooperation research < 49 times and performed further statistical analysis on the screened-out ten universities and think tanks. According to the analysis, the data of the ten universities and research institutes with the highest frequency of environmental research in Germany are as follows:

The data show that the top ten universities and research institutions engaged in environmental research in Germany are Helmholtz Center Environmental Research Center, Humboldt University, Dresden University of Technology, Berlin University of Technology, Technical University of Munich, Bremen University, Hamburg University, Bonn University, Aachen University of Technology, and Freiburg University. Among them, Helmholtz Environmental Research Center in Germany, Humboldt University in Germany, and Dresden University of Science and Technology in Germany rank among the top three in German environmental research, with a frequency of 99 times, 70 times, and 69 times, respectively, which dominates the research direction and research frontier of German environmental research, and University Bremen, University Hamburg, University Freiburg have a frequency of 59 times, 55 times, and 49 times. As a global stakeholder on the world map, academic institutions must expound on the conclusive findings to develop environmental feasibility. This reflects the joint venture of the socio-economic foundation pillars of Germany.

#### Cooperation and citation network analysis of researchers

After analyzing the cooperation between countries and institutions at the level of collaboration among scholars, the author set the cluster node as “author” and intuitively analyzed the cooperation and cited the situation of German scholars in environmental research, thus further presenting the collaboration among scholars in the field of environmental protection and essential author information in the last decade. We clicked “Visualize” to run CiteSpace without changing other parameters and, finally, received the following visualization diagram. The network analysis findings illustrate the authors' connectivity concerning environmental research being designated to investigate the spectrum of research that sags elements of the environment, causes, consequences, and concerns in the German circle. The results explain that the scholarly work is more concerned with the environmental domain, which is the roadmap for the German government and other institutions to design a collaborative pattern of global ecological success.

German scholars engaged in environmental research mainly included Jens Newig, Hans Wiesmeth, Henner Hollert, Claudia Pahlwostl, and Stefan Sagging, created by Martin Kowarsch (Martin). The scholars mentioned above mainly formed a research team led by ten scholars. From the map distribution, it can be further seen that, among the research teams led by these ten scholars, there were not only four large-scale research teams but also several small-scale cooperation networks with cross-cooperation. It is worth noting that the scholars mentioned above are all from top universities and scientific research institutions in Germany, including Helmholtz Center Environmental Research Center in Germany, Humboldt University in Germany, Dresden University of Science and Technology in Germany, Berlin University of Science and Technology in Germany, Munich University of Technology in Germany, the University of Bremen in Germany, Hamburg University in Germany, Bonn University in Germany, the Aachen University of Technology in Germany, and Freiburg University in Germany. These scholars reflect the main scientific research forces of environmental research in Germany. The abovementioned scholars' research is mainly supported by the Council of Europe Joint Research Center, the European Union, the German Research Foundation, the German Federal Ministry of Education and Research, and other institutions and foundations.

#### Analysis of research hotspots and research frontiers

To observe the research hotspots and frontiers in German-related environmental problems, the author selected “keyword” as clustering nodes in the research tool interface of CiteSpace. The time slice was set to 1 year to highlight the time changes of the analysis object in greater detail. Then, we clicked the “visualization” button to run CiteSpace, and the final software statistics chart is as follows.

**Figure 4:**
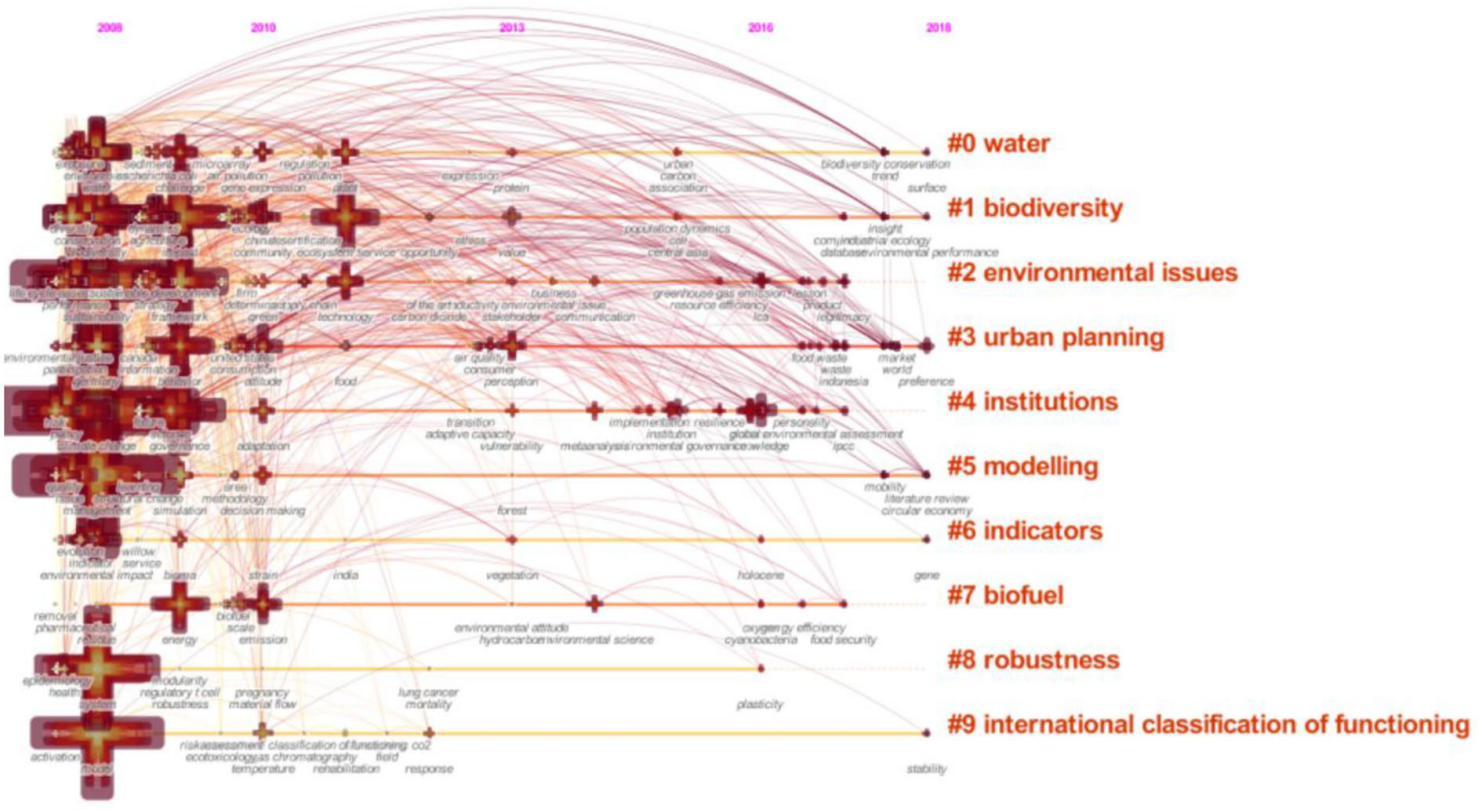
A time-shift diagram of high-frequency keywords and research frontiers of environmental problems in Germany. Source: This map was drawn by the author through CiteSpace.

According to the obtained analysis data, under this set of parameter setting conditions, “nodes” = 384 and “links” = 2,432 times; that is, a total of 384 keywords were included in this statistic, and there were 2,432 contacts among the above keywords. The author filtered the keyword information with a frequency of fewer than 47 times and finally collected 20 groups of keywords with a higher frequency in this field. The keywords that German scholars pay attention to environmental problems from high to low are climate change, sustainability, management, model, system, biodiversity, influence, framework, policy, governance, protection, performance, water, environment, behavior, science, ecosystem service, energy, problems, and risks (Figure 4).

**Figure 5:**
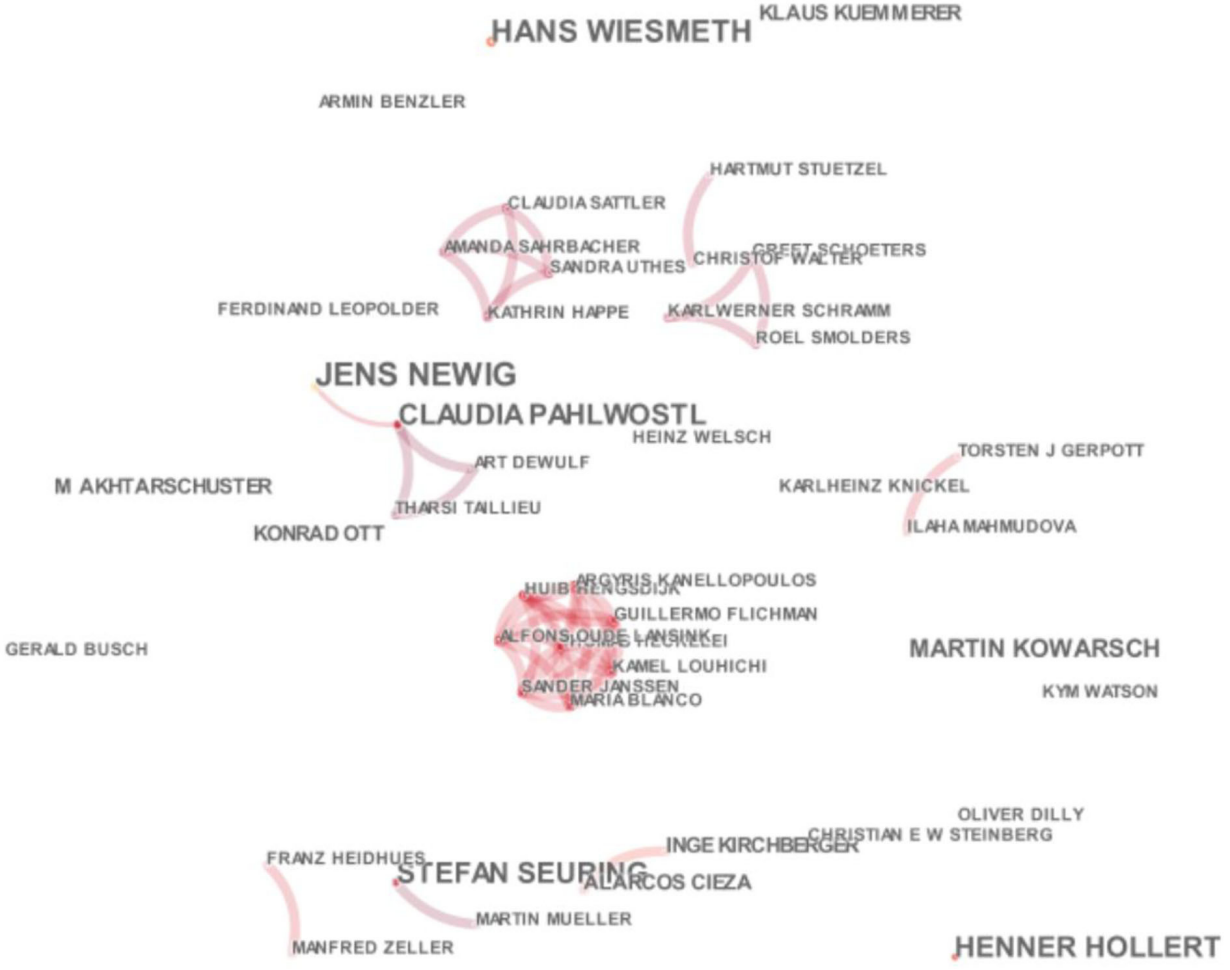
Cooperation network of German environmental researchers. Source: This map was drawn by the author through CiteSpace.

**Figure 6:**
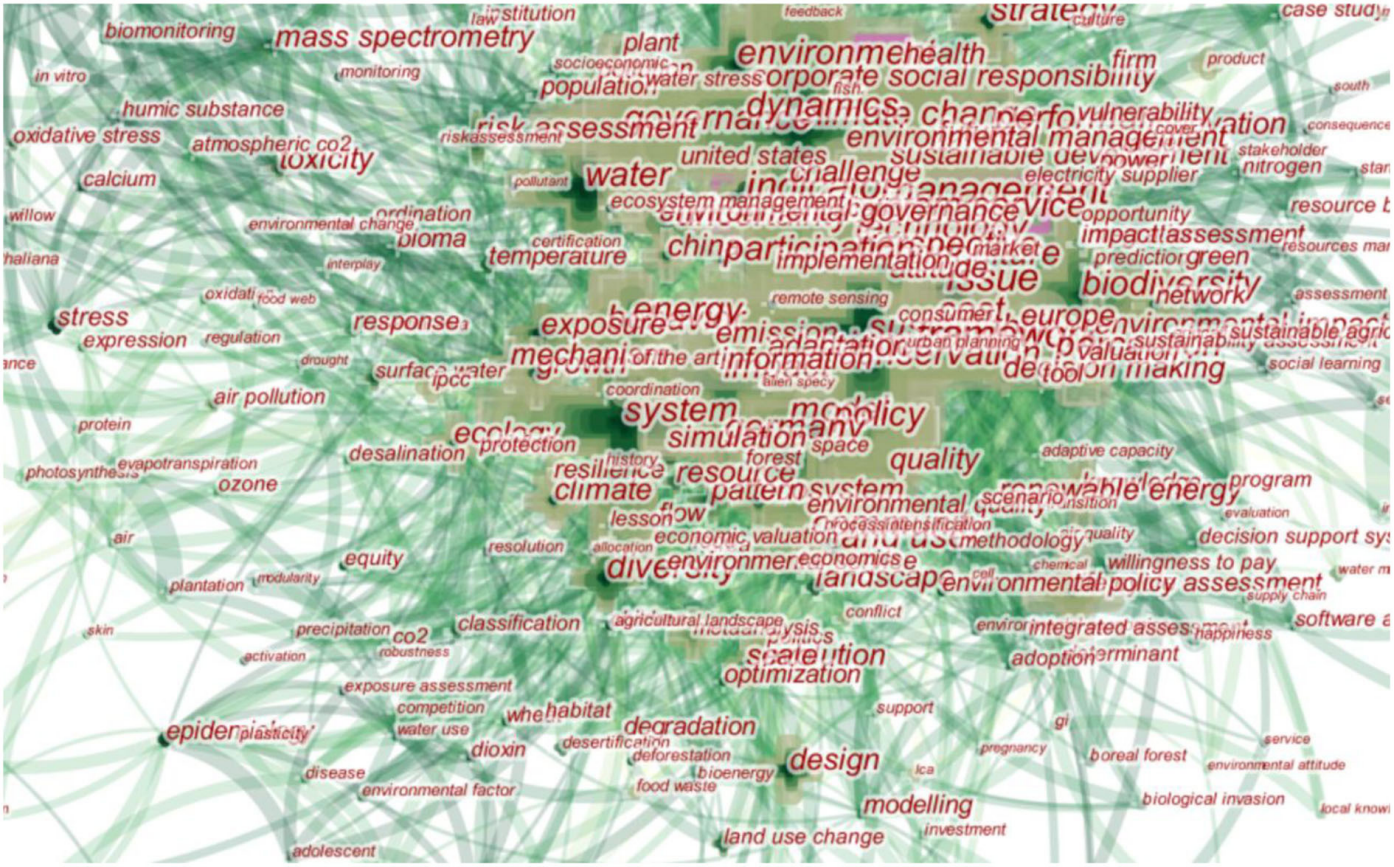
Keywords of German environmental research. Source: This map was drawn by the author through CiteSpace.

In Table 3, the 20 groups of keywords obtained from the abovementioned statistical screening represent the key points and important areas of continuous concern in the research of German environmental issues. To further analyze the frontier hot spots of environmental research in Germany, the author adjusted the view mode to a time zone view and finally obtained the time series map, as shown below (Figure 5). In the atlas, the top shows the changes in keywords and the frequency of keywords that German scholars pay attention to every year from 2008 to 2018. The larger the icon of the cluster node, the higher the attention displayed. On the right side of the atlas, the frontier of current research by German scholars and ten important words worthy of attention are displayed. The order is water resources, biodiversity, environmental problems, urban planning, institutions, indicators, biofuels, robustness, and international functional classification. It was found that, in recent years, the frontiers of German scholars' research on environmental issues have mainly focused on the fields of water resources and biodiversity, and the protection of water resources has always been an important topic in Germany. Germany has formulated laws and regulations on sewage treatment and rainwater treatment, established an Industry-University-Research cooperation platform, and realized the sustainable and rational utilization of water resources. In the field of biodiversity, Germany and China have had active dialogue and exchange. From 18 February 2019 to 22 Februrary 2019, the 11th Sino-German Symposium on Biodiversity and Ecosystem Services was held in Pu' er City, with in-depth discussions on such topics as the global biodiversity framework after 2020, natural capital accounting and national park system by the Ministry of Ecology and Environment of the People's Republic of China (2019). In addition, urban planning, biofuels, robustness, international functional classification, and other fields have also attracted German scholars' attention (see Figure 6).

**Table 3:** Top ten high-frequency keywords concerned by German environmental research.

**Serial number**	**Keywords (original words)**	**Frequency**	**Serial number**	**Keywords (original words)**	**Frequency**
1	Climate Change	177	11	Conservation	76
2	Sustainability	151	12	Performance	75
3	Management	145	13	Water	65
4	Model	119	14	Environment	64
5	System	117	15	Behavior	62
6	Biodiversity	98	16	Science	62
7	Impact	94	17	Ecosystem service	62
8	Framework	84	18	Energy	52
9	Policy	83	19	Issue	51
10	Governance	80	20	Risk	47

Source: This form was drawn by the author through CiteSpace.

## Conclusions

Germany has long been regarded as one of the countries with the best environmental protection and the most eco-environmental management experience. People attach importance to environmental issues, and the cooperation between China and Germany on environmental protection is an important starting point, whether it is for China-EU relations or for promoting the green cooperation of the Belt and Road Initiative to draw sustainable environmental gains together. It is vital for China and the European Union to jointly fulfill a big country's responsibility and promote the implementation of the global emission reduction task. To varying degrees, German and academic circles have affirmed China's emission reduction achievements as an important participant in the Paris Agreement and an important partner of the European Union. They have kept high expectations for China and Europe in the fields of global governance and environmental protection for a sustained future. With the rational use of the research tool CiteSpace, the author visually operated and analyzed the samples from the aspects of international cooperation, cooperation of scientific research institutions, cooperation networks of researchers, cited networks, research hotspots, research frontiers, and so on, based on the related research of German scholars on the environment collected by the Web of Science TM core collection database from 2008 to 2018, and finally came to the following conclusions.

Firstly, the author preliminarily clarified the status of German research on environmental issues, international cooperation, and scientific research cooperation from 2008 to 2018.

The second place in the German cooperation list is the United Kingdom, and the frequency of German-British cooperation is 321. However, Sino-German collaboration on environmental issues ranked 11th, and the frequency of the Sino-German association was only 126 times, which was still low. There was still much room for improvement. To better promote the economic recovery of both sides after the epidemic, China and Germany should uphold the concept of green development and strengthen green cooperation through high-level dialogue on the environment and climate. After the UK withdrew from the European Union, Germany's status as a major EU country became more important. In the European general election in 2019, Germany ranked first among the member countries with 96 seats, especially because the influence of the Green Party group is inseparable from the German Green Party. To further strengthen China-EU relations, we must improve cooperation with Germany in various aspects.

Third, Germany's research on environmental issues is mainly led by German universities and research institutions, including Helmholtz Central Environmental Research Center, Humboldt University, Dresden University of Science and Technology, Berlin University of Science and Technology, Munich University of Technology, University of Bremen, Hamburg University, Bonn University, Aachen University of Technology, Freiburg University, and other universities and research institutions, including Helmholtz Central Environmental Research Center, Humboldt University, and Germany. German scholars engaged in environmental research mainly include a research team composed of Jane Newweig, Hans Weismenz, Henna Holt, Cordia Pelle Vast, Stefan Eli, Martin Kovac, Akutas Hurst, Klaus Cuomo, Connard Walter, and Alai Seza. The scholars mentioned above mainly set up four large-scale research teams and several small-scale cooperation networks with a cross. The council of Europe Joint Research Center, the European Union, the German Research Foundation, the German Federal Ministry of Education and Research, and other institutions and foundations provided corresponding financial support and funding for the scholars mentioned above, effectively promoting the research of environmental problems in Germany. In March 2019, the German Federal Ministry of Education and Research, the German Ministry of Education and Research put forward the “New Initiative of Biodiversity Conservation Research,” expressing the determination to protect global biodiversity. In this regard, China has always attached great importance to biodiversity protection and has actively participated in global biodiversity governance. At the second Belt and Road Initiative international cooperation summit forum, Chinese President Xi Jinping emphasized that “we should take green as the background, promote green infrastructure construction, green investment, and green finance, and protect our common living homeland.” Both sides have played important roles in promoting the realization of the post-2020 global biodiversity framework. In addition, we need to pay attention to the role of many social organizations in promoting public welfare. For example, the Sino-German Environmental Energy Center is committed to promoting exchanges and cooperation between the two sides in the environmental and energy fields. At the same time, promoting advanced environmental protection technologies and popularizing advanced European environmental protection ideas are two different things. Active participation is vital in Sino-German environmental research cooperation projects, such as the Sino-German clean water project and Horizon 2020.

Fourth, regarding environmental problems, 384 keyword frequencies have been included in this statistical data. The top 20 keywords with the closest attention and frequency were climate change, sustainable development, management, mode, system, biodiversity, influence, framework, policy, governance, protection, performance, water resources, environment, behavior, science, and ecosystem services. After further adjusting the view mode to time zone view, it was found that the current frontier keywords of German environmental research mainly include water resources, biodiversity, environmental problems, urban planning, institutions, models, indices, biofuels, robustness, and international functional classification. Many studies on environmental problems in Germany are focused on the fields of water resources and biodiversity. In addition, urban planning, biofuels, and robustness have also attracted wide attention from German academic circles.

To sum up, by analyzing the relevant literature data collected by German scholars on environmental issues in the Web of Science TM core collection database, the author has derived 2,714 research results. These research results have great representativeness and coverage. They can truly reflect the current international cooperation in environmental research in Germany, the collaboration of scientific research institutions, the cooperation network of researchers, cited networks, research hotspots, research frontiers, and so on. After the introduction of “Industry 4.0” in Germany in 2013, environmental issues became a priority for the government and private industry alike, and significant progress was achieved toward protecting the environment in the country. In the future, there is still much room for development in the cooperation between China and Germany on environmental protection. To optimize and sustainably develop China's environmental protection policies, the country can keep abreast of the latest research achievements, research frontiers, and trends in the field of environmental protection in Germany, strengthen academic exchanges between China and Germany, increase the number of scholars and international students sent to Germany, and promote the continuous progress of China's environmental protection industry. According to the data of the German Federal Statistical Office, despite the tremendous negative impact of the epidemic, the bilateral trade volume between China and Germany increased by 3% last year to about 212.1 billion euros. In 2021, the fifteenth meeting of the Conference of the Parties to the Convention on Biological Diversity was held in Kunming, China. Both China and Germany should shoulder the responsibility of protecting the environment by strengthening innovative cooperation in climate change and enhancing the level of Sino-German collaboration on the environment and climate.

### Study implications

First, in relation to the significance of this research, the current study compiles the global connection of environmental research knowledge supplied by research stakeholders. Second, this research is a worthy addition to the canon of knowledge, having been published in the prestigious peer-reviewed journals of academic powerhouses in industrialized nations. Third, this study has inducted five clusters of environmental research contributions into the field of research. To rethink and understand environmental concerns, we visualize the environmental pillar of sustainability, which is the very existence of human life in developed countries. The connectivity of economic and social components of sustainability is complemented by environmental sustainability. This is what this field of work considers from the well-reputed research publisher in the global arena. This study's application of a global perspective in areas such as water resources, biodiversity, environmental problems, urban planning, institutions, indicators, biofuels, robustness, and international functional classification represents a timely contribution that recognizes research concerns in Germany that are working to transform development into sustainable development. Followed by the next dimension, this study reveals critical keywords studied in the past concerning environmental research. The bilateral cooperation of China and Germany on the environment and developing new energy sources are practical steps. There is a Sino-German ecological park in Qingdao. The Chinese Ministry of Science and Technology is working with its German counterpart to advance electric bikes, electric vehicles, and hybrid vehicle R&D in Beijing and Shanghai. In terms of managerial contributions, the study urges organizational stakeholders to promote policies that would result in a greener environment to promote sustainable development. The global partnership between heavily industrialized countries must be seen in connection to environmental sustainability through shared experiences. The consideration of academic studies on environmental research must be integrated into strategic management plans to ensure sustainable development is reported in scientific literature.

### Study limitations

This study fully uses CiteSpace tools to analyze the environmental research situation in Germany and draw corresponding conclusions. The diversity of data sources, the interests or preferences of researchers, and the research methods' applicability all constitute the research process's limitations and conclusions. The limitations of the research in this paper are mainly as follows:

Firstly, CiteSpace, as the research tool of this study, although it can show the relevance of the analyzed objects, cannot provide quantitative indicators of the degree of relevance. Hence, the whole research is still in the qualitative analysis phase.

Secondly, the data lag of the paper mainly obtains the research data through the network and retrieval database. There need to be more current achievements to support the research. Thirdly, China and Germany have different national conditions, and the applicability of the research results is limited. Germany is a developed country that has entered the stage of post-industrialization. Its thinking and policies on green industry and green consumption are not necessarily suitable for China's current national conditions and the development stage. This means that the research conclusions and thoughts in this study are more of a useful reference for China's future green development than a means that can be directly used.

### Further directions

In future research, comprehensive resources can be helpful in the utilization of the results of German environmental research so that it can be adapted to China's national conditions and promote in-depth cooperation between China and Germany in the fields of low-carbon development and green industry. Green practices and digital technologies are concrete measures of such collaboration. With the unprecedented high temperatures and drought in the Northern Hemisphere in 2022, people are already feeling the effects of global warming. Research and decisive action on environmental issues are urgent. Environmental research collaboration between China and Germany should also address this dire scenario. Green practice involves production and consumption to operate in developmental domain of anthropology. In production, digital technology significantly enhances management efficiency and reduces wasteful resource use and management-related waste. Green practices will transform people's eating habits, social activities, transportation, etc., in the consumer sector. These subjects can serve as future directions for environmental studies study.

## Data availability statement

The original contributions presented in the study are included in the article/supplementary material, further inquiries can be directed to the corresponding authors.

## Author contributions

WZ: conceptualization and data collection. BT: data management: FW: project administration. HT: data analysis. HZ: paper drafting. CW: validation and conducted the survey. MT: methodology and reviewing. All authors have read and agreed to the published version of the manuscript.
